# Calibration of the Leg Muscle Responses Elicited by Predictable Perturbations of Stance and the Effect of Vision

**DOI:** 10.3389/fnhum.2016.00419

**Published:** 2016-08-30

**Authors:** Stefania Sozzi, Antonio Nardone, Marco Schieppati

**Affiliations:** ^1^Centro Studi Attività Motorie (CSAM), Fondazione Salvatore Maugeri (IRCCS)Pavia, Italy; ^2^Posture and Movement Laboratory, Physical Medicine and Rehabilitation, Fondazione Salvatore Maugeri (IRCCS)Veruno, Italy; ^3^Department of Translational Medicine, University of Eastern PiedmontNovara, Italy; ^4^Department of Public Health, Experimental and Forensic Medicine, University of PaviaPavia, Italy

**Keywords:** moving platform, balancing behavior, adaptation, kinematics, EMG, reflex and anticipatory response, vision

## Abstract

Motor adaptation due to task practice implies a gradual shift from deliberate control of behavior to automatic processing, which is less resource- and effort-demanding. This is true both for deliberate aiming movements and for more stereotyped movements such as locomotion and equilibrium maintenance. Balance control under persisting critical conditions would require large conscious and motor effort in the absence of gradual modification of the behavior. We defined time-course of kinematic and muscle features of the process of adaptation to repeated, predictable perturbations of balance eliciting both reflex and anticipatory responses. Fifty-nine sinusoidal (10 cm, 0.6 Hz) platform displacement cycles were administered to 10 subjects eyes-closed (EC) and eyes-open (EO). Head and Center of Mass (CoM) position, ankle angle and Tibialis Anterior (TA) and Soleus (Sol) EMG were assessed. EMG bursts were classified as reflex or anticipatory based on the relationship between burst amplitude and ankle angular velocity. Muscle activity decreased over time, to a much larger extent for TA than Sol. The attenuation was larger for the reflex than the anticipatory responses. Regardless of muscle activity attenuation, latency of muscle bursts and peak-to-peak CoM displacement did not change across perturbation cycles. Vision more than doubled speed and the amount of EMG adaptation particularly for TA activity, rapidly enhanced body segment coordination, and crucially reduced head displacement. The findings give new insight on the mode of amplitude- and time-modulation of motor output during adaptation in a balancing task, advocate a protocol for assessing flexibility of balance strategies, and provide a reference for addressing balance problems in patients with movement disorders.

## Introduction

Practice improves movement kinematics and diminishes the accompanying metabolic and cognitive cost. Motor adaptation implies a gradual shift from a more conscious control of behavior to automatic processing, which is less resource- and effort-demanding, and produces a gradual modification of the behavior that makes it more fit under the conditions of its environment (Shadmehr et al., 2010). Adaptation is common to both voluntary movements, where repetition and task practice improve performance (Lotze et al., 2003), and movements in which automatism prevails (Saling and Phillips, 2007) as in locomotor (Prokop et al., 1995) and postural tasks (Nashner, 1976). Balance perturbations can be of very different nature, from displacement of the base of support (Nashner, 1976; Horak and Nashner, 1986) to vibratory proprioceptive (De Nunzio and Schieppati, 2007; De Nunzio et al., 2008) and galvanic vestibular stimulation (Fransson et al., 2007; Tjernström et al., 2010a). Berger et al. (1992) and Dietz et al. (1993) described leg muscle activity and biomechanical patterns in subjects standing on an unstable support basis, such as a treadmill moving backward and forward. Their pioneer study suggested that the aim of the control process is to stabilize the position of the body’s center of gravity relative to the feet, while the forces acting on the body during the treadmill movements are minimized.

Balance perturbations delivered by repeated sinusoidal translations of the support base have been employed frequently in both control subjects and patients, in order to investigate balancing behavior under dynamic conditions, as opposed to quiet stance. Within the frame of a reproducible general pattern, an ample range of variability has been observed for successive cycles of the same perturbation, suggesting flexibility of dynamic postures (Schieppati et al., 2002; Cappa et al., 2008; Kennedy et al., 2012). Clear-cut differences in balancing behavior have also been observed in the presence or absence of vision (Buchanan and Horak, 1999; Corna et al., 1999; Fujiwara et al., 2006; Schmid et al., 2011). For instance, when vision is gradually degraded experimentally, good visual acuity strongly reinforces a “head-fixed-in-space” behavior, while poor vision and no vision rather produce a “head-moving-with-platform” displacement (Schmid et al., 2007, 2008). Notably, aging is associated with reduced head stabilization in the absence of vision, particularly for high-frequency platform translations (Nardone et al., 2000). The balancing behavior is fairly resistant to abnormal proprioceptive noise obtained by postural muscle vibration (De Nunzio et al., 2005) and is hardly affected in patients with neuropathy and with Parkinson’s disease (Nardone and Schieppati, 2006; Nardone et al., 2006, 2007), pointing to significant intervention of feed-forward mechanisms.

In the above cited reports, the first few cycles of the sequence of oscillations were deliberately excluded from the analysis in order to get rid of the initial large EMG activation bursts and body movements elicited by the supposed startling effect of the onset of the perturbation (Nonnekes et al., 2015), and to describe and measure the adapted behavior at steady state. The question of the initial EMG adaptation had been addressed with a sequence of discrete impulsive perturbations by Keshner et al. (1987), who suggested that adaptation is due to a generalized habituation in the postural control system. Fujiwara et al. (2007) described adaptation of the balancing strategy to continuous floor oscillation. By recording event-related potentials, reflecting cortical activation by sensory information related to the postural disturbance, they suggested that attention to information processing decreases with adaptation (Fujiwara et al., 2012, 2016). Mierau et al. (2015) also reported adaptation of the negative cortical potential and reduced muscle co-contraction during a prolonged balancing task under critical standing-balance condition. Notably, at the beginning of the perturbation series, the extra facilitatory effect from higher centers on the spinal motoneuronal pools gradually vanishes as automatisms prevail (Solopova et al., 2014).

Siegmund et al. (2008) advised that the large muscle response to a novel transient perturbation consists of combined postural and startle responses. On the other hand, Oude Nijhuis et al. (2009) and Allum et al. (2011) reported on the response to the first perturbing trial of a sequence, and compared that response to a startle reaction elicited by an acoustic stimulus. They highlighted the substantial differences between the “true” startle reaction and the response to the first trial of a balance perturbation, and concluded that future studies should no longer discard the latter response, but routinely include it in the analysis (Tang et al., 2012).

Short- and long-latency leg muscle reflexes are elicited by the displacement of the body segments, and produce balance correcting effects (Horak et al., 1989; Nardone et al., 1990). Proactive strategies (Massion, 1992; Bouisset and Do, 2008) are also produced to counteract the balance perturbations elicited by the platform displacement reversal (Rogers et al., 2003; Jacobs and Horak, 2007). These anticipatory postural adjustments may not necessarily be optimally tuned to the complex combination of active and passive body movements from the beginning of the perturbation sequence (Aruin et al., 1998, 2015). In the assumption that both reflex responses and proactive strategies are contributing to the balancing behavior on the continuously translating platform (Laessoe and Voigt, 2008) and that both undergo adaptation with the repetition of the perturbation cycles (Dietz et al., 1993; Taube et al., 2007; Kennedy et al., 2012), we set out to record activity of the leg flexor and extensor postural muscles and body kinematics. Our attempt was to better identify the muscle responses and the potentially different adaptation pattern thereof, and to assess any relationship between the adaptation of muscle activity and the balancing behavior by using support surface translation stimuli. We expected to get an answer to the following questions. What is the time course of adaptation? Is adaptation common to both muscles acting on the ankle joint? Is this process accompanied by modulation of co-contraction rate of both antagonist muscles? Is it similar for muscle activities having a presumed different origin (reflex vs. proactive)? Does adaptation imply amplitude modulation of EMG bursts, or does their latency also change over time? Is there a correspondence between adaptation of leg muscle activity and changes in the position of the center of mass (CoM)? To what extent does vision affect adaptation? What are the functional advantages of adaptation?

## Materials and Methods

### Subjects and Task

Ten healthy subjects (5 males and 5 females, mean age 30.8 years ± 6.3 SD, height 173.7 cm ± 5.3 SD, weight 66.2 kg ± 10.9 SD) stood with bare feet spaced about 10 cm apart on a mobile platform, translating in a sinusoidal way in the antero-posterior (A-P) direction at 0.6 Hz and 10 cm amplitude. They were not aware of the onset of the movement of the platform, which started pseudo-randomly within 5–10 s from the acquisition onset (known to the subjects). Subjects wore soundproof earphones in order to mask the noise made by the platform. Each acquisition was composed of three parts: subjects stood on the still platform (5–10 s); the platform made 59 consecutive oscillation cycles (total duration 100 s); the platform stopped and subjects stood on the still platform for 5 s more. Subjects performed one single such trial with the eyes closed (EC), and one trial with the eyes open (EO) in a separate session after 5–7 days. Felt-tip pen marks on the skin helped to place the electrodes in the same position during the two test conditions. No randomization was performed. In parallel experiments, a second group of naive subjects was studied (*n* = 10; 4 males and 6 females, mean age 25.5 years ± 2.9 SD, height 171.8 cm ± 9.2 SD, weight 68.6 kg ± 16.3 SD), to which EO condition only was administered. This allowed checking any persistence of adapted behavior due to the EC trial preceding the EO trial in the former group of subjects. All subjects were naive to the experimental procedures or to any balance-oriented experiments and all succeeded in performing the trials. Experiments were performed in accordance with the Declaration of Helsinki. The institutional ethics committee (Central Ethics Committee, Fondazione Salvatore Maugeri, approval number # 905 CEC) specifically approved the study and the consent procedures, which were carried out with the adequate understanding and written informed consent.

### Data Acquisition

Kinematic data were recorded by means of an optoelectronic device (Smart-D, BTS, Italy). For computation of CoM and orientation in space of the body segments, 19 reflective markers were placed bilaterally on these body positions (Winter, 2009): vertex and lateral head, acromion, C7, L5, anterior superior iliac spinae, greater trochanter, lateral epicondyle of the femur, lateral malleolus, heel and forefoot (dorsally, about over the 1st metatarso-phalangeal joint). Subjects’ arms were folded not to interfere with marker capture. The marker positions in space were recorded by 12 cameras at 140 Hz and stored in a PC for off-line analysis. For subsequent analysis, the kinematic data were resampled at a frequency of 1000 Hz after linear interpolation by the BTS proprietary software, in order to display both kinematic and EMG data on the same scale.

EMG was recorded by pairs of surface electrodes placed, for both legs, over the muscle bellies of Tibialis Anterior (TA) on the anterior aspect of the upper third of the leg, and of Soleus (Sol) posteriorly, 5 cm below the insertion of the gastrocnemii in the Achilles’ tendon. The distance between the recording leads was 1.5 cm. EMGs were wirelessly recorded (Freeemg, BTS, Italy) at 1000 Hz. Signals were filtered with a high-pass filter (cut-off 50 Hz), full-wave rectified and then filtered with a low-pass filter with a cut-off frequency of 200 Hz. EMG signals were acquired through the same Smart-D system, and were synchronized with the kinematic data.

The EMGs of TA and Sol of both legs were also recorded in each subject during *ad hoc* trials in which subjects produced bursts of maximal activity, for each muscle separately, before each EC and EO trial. For each muscle and leg, three maximal isometric contraction efforts, each lasting 2–4 s, were performed. For TA, during standing, the foot was blocked to the ground preventing foot dorsiflexion. For Sol, while subjects were sitting on a chair, the knee was blocked against a resistance to keep ankle and knee angles at about 90°. The level of EMGs activity recorded during the balancing trials were expressed as percent of the maximal voluntary activity of the respective muscles.

### Data Analysis

#### EMG Activity and Burst Identification

We offline separated the cycles of the platform perturbation by a software developed using Labview (National Instruments, Austin, TX, USA), where each cycle began with the platform forward translation. For a clearer representation of what happened during each cycle, in the Figures 4–9 a time-window starting 0.2 s before the initial forward movement of the platform and lasting 2.5 s is depicted, whereas the analyses were made on a time-window having a duration equal to the oscillation cycle (1.7 s) starting from the backward-most platform position.

The mean value of TA and Sol activity within each of the successive cycles was calculated in each subject. In addition, the traces of the rectified TA and Sol activity of all consecutive cycles were averaged. Different bursts of activity were regularly present on the TA and Sol mean traces. The time-windows, in which TA or Sol bursts were consistently present, are highlighted by shaded areas in Figure 4. For TA, we classified: (a) a reflex response, the first part of which occurred at about 80 ms after the beginning of the forward translation of the platform and lasted about 100 ms; this response was named medium-latency response (MLR), following Schieppati and Nardone (1995) and Nardone and Schieppati (1998); (b) a second part of the reflex response occurred at about 200 ms after the forward platform translation and lasted about 100 ms; this was the long-latency response (LLR; Nardone et al., 1990); (c) a burst just around the anterior platform turn-around point, starting about 700 ms after the forward translation and lasting about 200 ms, was arbitrarily named fall-preventing response (FPR) and (d) a burst roughly in correspondence with the period of backward platform movement, starting at around 1.2 s after the forward translation and lasting around 300 ms; this was arbitrarily named proactive response (PAR), since it brakes the CoM backward displacement just prior to the forward platform movement of the subsequent cycle. For Sol, the bursts were almost superimposable to those of TA when the Sol trace was shifted by $\frac{1}{2}$ cycle, so that stretch and anticipatory responses would match the corresponding platform displacement (forward for TA and backward for Sol). Hence, following the same criteria used for TA and referring to the onset of forward translation, we identified: (a) a burst before the peak of the forward excursion, starting about 300 ms after forward translation and lasting about 300 ms (PAR), this response mainly occurs during Sol shortening, and would correspond to the TA PAR; (b) a double-peak reflex response, occurring at about 800 ms after the forward translation (at the beginning of the backward platform displacement) and lasting about 100 ms; in the Sol, a short-latency stretch response is evoked immediately prior to the MLR (hence, this burst was named SLR + MLR); (c) a burst in correspondence with the backward platform movement, starting at around 1.1 s after the forward translation and lasting around 100 ms (LLR); and (d) a burst after the peak of backward excursion, starting about 1.5 s after the forward translation and lasting about 100 ms (FPR). Then, for each cycle of each subject, the actual onset and termination of each response was visually identified by means of a custom-made Labview software on the display of each EMG trace. The response identification was aided by the superimposition of the traces of the homonymous muscles of both legs. Two experimenters (SS and MS) agreed on the identification of the burst onset and termination. In the subsequent analysis, the onset latency of each TA response was referred to the onset of platform forward movement, and for Sol to the onset of platform backward movement.

The area (time-integral) of the rectified TA and Sol muscle EMG measured within the time-interval defined by onset and end of each response was expressed as a percentage of the area of the EMG corresponding to the maximal voluntary contraction, calculated on an equal time-interval as the individual response bursts. The area of the TA and Sol bursts were plotted against the ankle angular velocity measured 50 ms before the onset of the respective TA or Sol burst in order to check the hypothesis that the response is related to muscle stretch and represents a short-latency response to muscle lengthening. The 50 ms interval was arbitrarily selected for all responses, based on the presumed “average” latency of the TA and Sol reflex activation by muscle stretch, and assuming that the muscles had no slack due to their background activation.

#### Kinematics

The CoM was computed according to the Winter (2009) protocol by a software developed in Matlab (MathWorks Inc., Natick, MA, USA). In order to infer changes in muscle length and velocity occurring during the perturbation cycles, the ankle angle of both legs was computed from the position in space of the markers placed on the lateral femoral condyle, lateral malleolus and forefoot. The changes in foot dorsum deformation were estimated from the height of the marker placed on malleolus. The marker traces were filtered with a low-pass filter (cut-off 3 Hz). The velocity of ankle angular variation was the derivative of the ankle angle. Positive velocities corresponded to TA stretch (or Sol lengthening).

For each subject, the standard deviation of all the values of the A-P displacement trace of CoM and head vertex was calculated for the entire duration of the platform perturbation, and considered a global index of displacement amplitude (small and large standard deviations pointing to stable and unstable segment position in space, respectively; Corna et al., 1999). For each subject and oscillation cycle, a cross-correlation (CC) analysis was performed between the traces of platform and CoM A-P displacement (both referred to the laboratory space). The CC coefficient (*R*) at time lag = 0 s was calculated by means of the CC routine of the software Origin (OriginLab Corporation, Northampton, MA, USA). A positive coefficient indicates in-phase displacement of CoM and platform, a negative coefficient indicates anti-phase displacement. The time lag was the time interval at which the absolute value of R was maximum. The 95% confidence interval of the highest CC value was used to assess if the time lag between platform and CoM displacement was statistically significant (Li and Caldwell, 1999). A positive time lag indicates that CoM movement lagged the platform movement.

The CoM back-and-forth displacement with respect to the malleolus was also plotted against the ankle angle and the coefficient of determination (*r*^2^) of the regression line was calculated for each subject and successive cycle.

#### Adaptation Rate Assessed by Exponential Fit

In order to grossly quantify the adaptation process, we fitted the relevant data with an exponential function (*y* = A + Be^−t/τ^). To this aim, the exponential-fit routine of the software Origin was used, τ (tau) being the time-constant, A the value at steady state (asymptote), A + B the intercept with the ordinate (Sozzi et al., 2011). A, B and τ parameters were computed by using the Levenberg-Marquardt algorithm. Using this procedure, we fitted over time the mean CoM A-P position, the time lags between platform and CoM displacement. The changes in the relationship between CoM displacement and ankle angle identified by the *r*^2^ coefficient of the regression lines were also fitted in the same way. The mean EMG activity of TA and Sol of each cycle was plotted as a function of the successive cycles: these values generally exhibited a rapid initial variation and a trend to plateau with time. Of note, the exponential fit was made on the entire sequence of cycles, including the first one. The time, at which steady state was reached, was estimated by 3*τ, which corresponds to a reduction to less than 5% of the initial value.

In addition, we fitted a double exponential function (*y* = Ae^−t/τ1^ + Be^−t/τ2^ + C) to the area data of the identified bursts of the successive cycles in order to take into account the possibility that a dual process (Huberdeau et al., 2015) featuring a complex evolution over time underpins the adaptation of both reflex and proactive bursts. *τ*_1_ and *τ*_2_ were the time-constants (expressed in cycle number), C the value at steady state, A + B + C the intercept with the ordinate. The values of the asymptote and of the intercept at the second oscillation cycle of the fitted exponentials (therefore excluding the “first-trial effect”) served to assess the amplitude adaptation of the EMG responses. When the software did not converge to the imposed exponential, we simply interpolated the data with a linear fit, and estimated the amplitude of adaptation by the difference between the values of the fitted line at the second and last cycle.

#### Assessment of the TA-Sol Co-Contraction Pattern

In order to quantify the possible co-contraction of TA and Sol muscles and its variation over time, for each cycle the instantaneous rectified EMG activity of the two muscles of the right leg was plotted one against the other. For each subject and cycle, we defined a co-contraction index as follows: each value of the level of TA activity was multiplied by the corresponding value of Sol activity, and all products were averaged. Each co-contraction index of each cycle was then identified by a variable *k* (the mean value of all products within the cycle). Hence, for each subject, the *k* values across all successive cycles were fitted with the exponential function *y* = A + Be^−t/τ^, in order to assess any change over time of the co-contraction pattern.

#### Statistical Analysis

Statistical computations are listed below in the order of appearance in the “Results” section. All variables had normal distribution, as tested by the Kolmogorov-Smirnov test. Systematic effects (EO vs. EC, TA vs. Sol) often produced substantially different data variability, as tested by Levene’s test. However, since the coefficients of variations were remarkably constant across conditions, the log transformation was applied (Lison, 1960) prior to applying parametric statistics.

The mean activity across cycles of TA and Sol was compared by a 3-way repeated measures ANOVA with muscles (TA and Sol), visual condition (EC and EO) and leg (right and left) as factors. Across subjects, the time-constants of the mean activity values of TA and Sol were compared by a 2-way repeated measures ANOVA, with muscles and visual condition as factors. The mean EMG activities at steady state (the asymptotic value of the exponential fit) were compared by a 2-way repeated measures ANOVA, with muscle and visual condition as factors.

The time-constants of EMG adaptation (TA and Sol) between the group that received the EO perturbations only and the group that received the EO after the EC perturbations were compared, separately for the two muscles, with a Student’s *t*-test.

Three-way repeated-measures ANOVAs, with muscle, vision, response type (MLR or SLR + MLR, LLR, FPR and PAR) as factors, were used to compare: onset latency, duration and area of TA and Sol response bursts, and ankle angular velocities. Four one-way ANOVA were used to assess differences in the mean latencies of various TA EC responses across cycles.

The changes in the area of TA and Sol responses across the successive cycles were interpolated with a double exponential function. When the algorithm converged to the exponential model, the time-constants thus obtained (*τ*_1_ and *τ*_2_) were compared by a 4-way repeated-measures ANOVA with *τ*_1_ and *τ*_2_, muscle, visual condition, response type (MLR or SLR + MLR, LLR, FPR and PAR) as factors.

To estimate the adaptation in burst amplitude, the differences between the intercept values at the second oscillation cycle and the asymptotic values were compared with a 4-way repeated-measures ANOVA with EMG level at second cycle and steady-state, muscle, visual condition and response type as factors.

The standard deviations of CoM and of head periodic displacement (arbitrarily taken as a global index of segment instability) were compared with a 2-way repeated measures ANOVA with variables (CoM and head) and visual condition (EC and EO) as factors.

The time-constants obtained by fitting the exponential functions to the CC coefficients and to the time-lag between CoM and platform across cycles were compared between EO and EC by paired Student’s *t*-test. The time-constant of the changes over the successive cycles of the *r*^2^ coefficient of the lines best fitting CoM against ankle angle and that of the parameter *k* (indicating co-contraction) were compared with a 2-way repeated measures ANOVA with variables (time-constants of *r*^2^ and *k*) and visual conditions as factors. For all ANOVAs, the *post hoc* analyses were made with the Fisher’s LSD test. The software package used was Statistica (StatSoft, Tulsa, OK, USA).

## Results

### Changes in EMG and Kinematics Over Time

**Figure 1:**
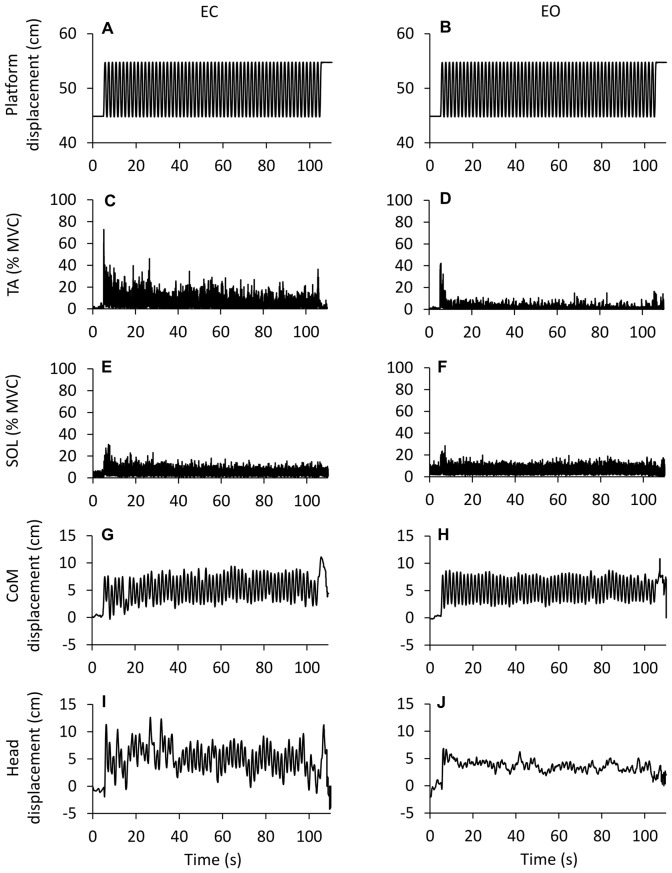
**Changes in EMG and kinematics over time.** Grand mean (*n* = 10 subjects) of the traces recorded during eyes closed (EC) and eyes open (EO) conditions. **(A,B)** show the platform sinusoidal translation (10 cm, 0.6 Hz) in the antero-posterior (A-P) direction (forward upwards). The first cycle begins after 5 s from the acquisition onset, the last cycle ends at 105 s. **(C–F)** show the grand mean of the rectified EMG traces of the Tibialis Anterior (TA) and Soleus (Sol) expressed as percent of the maximal voluntary contraction. There is a progressive reduction in muscle activity EC and EO. TA activity is much smaller EO than EC (not so for Sol). **(G,H)** Displacement of center of mass (CoM). **(I,J)** Displacement of head (marker on the vertex). The CoM back-and-forth displacement is similar EC and EO, whereas the head back-and-forth displacement is larger EC than EO. The larger deflections of the EMG and kinematic traces at the very end of the acquisition are connected with the sudden halt of the platform.

Figure 1 illustrates the adaptation phenomenon: the top trace **(A,B)** is the platform periodic displacement, identical for the EC and EO condition. Figures **(C–F)** show the grand mean (of the 10 subjects, who performed the EO after the EC trial) of the rectified traces of the TA and Sol muscle activity over the entire perturbation period. Displacements of CoM and head vertex are reported for EC and EO in **(G–J)**, respectively. A progressive reduction in the TA activity was obvious with EC **(C)**. Sol activity diminished rapidly to then remain sustained until the end of the acquisition period **(E)**. TA activity was much smaller under EO than EC condition, both at the beginning and at steady-state **(D)**, which was reached within very few cycles. Conversely, the level of Sol activity with EO **(F)** was broadly similar to that recorded with EC. CoM displacement was consistent from the beginning to the end of the translation cycles, and was similar under EC and EO condition. Still, the head back-and-forth displacement was much larger and more variable for EC than EO. Overall, these patterns of EMG and kinematic changes were common to all subjects.

### TA and Sol Activity Across Successive Cycles: Levels and Time-Constants

**Figure 2:**
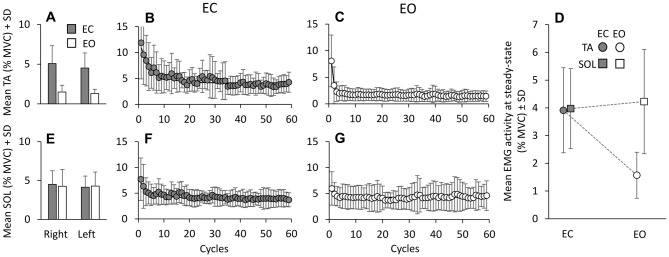
**Mean TA and Sol activity.** Mean muscle activities, across cycles and subjects in EC (gray bars) and EO (white bars) conditions, are almost the same between legs, for TA **(A)** and Sol **(E)**. The mean activity of TA **(B,C)** and Sol **(F,G)** are calculated for each platform cycle, EC and EO. With EC, both TA and Sol activities gradually decrease to steady state. The exponential functions fitted to the EC data are *y* = 4.15 + 6.77e^−t/5.1^ for TA and *y* = 4.09 + 2.87e^−t/4.3^ for Sol. With EO, both TA and Sol activities reach steady state very soon. The functions best fitting the mean muscle activities with EO are: *y* = 1.63 + 6.42e^−t/0.8^ for TA and *y* = 4.26 + 1.71e^−t/0.97^ for Sol. **(D)** shows that the mean Sol activity level at steady state is similar EC and EO, while TA is greatly reduced EO.

The mean activity of TA and Sol was averaged cycle by cycle across subjects, for EC and EO. It was similar between right and left leg, both EC (gray bars in **A** and **E**) and EO (white bars in **A** and **E**) (*F*_(1,9)_ = 2.16, *p* = 0.17; Figure 2). Hence, further analysis was performed on the mean EMG values of the two legs. Figure 2 also shows the grand mean of the EMG activity of each of the successive perturbation cycles, for TA **(B,C)** and Sol **(F,G)** during the EC and EO conditions. With EC, a gradual decrease in TA activity is obvious **(B)**. The exponential curve fitted to the data points has a time-constant of about 5 cycles (*τ* = 5.1 cycles); therefore, after about 15 cycles (~3*τ), i.e., about 25 s considering that each cycle lasts 1.7 s, TA activity reached a value close to the asymptotic value (steady-state). A gradual decrease was also present for Sol activity with EC (**F**, *τ* = 4.3 cycles). The mean values of the time-constants of the exponentials fitted to the profiles of TA and Sol activity of each subject were 6.5 ± 4.8 cycles (TA) and 5.9 ± 5.6 cycles (Sol) (not shown in Figure). With EO, both TA **(C)** and Sol **(G)** activities diminished rapidly and reached steady state very quickly (*τ* = 1.1 ± 0.7 cycles, TA; 3.4 ± 3.7 cycles, Sol). ANOVA on the time-constants of TA and Sol, after log transformation, showed a difference between visual conditions (*F*_(1,9)_ = 23.75, *p* < 0.001), no difference between muscles (*F*_(1,9)_ = 3.23, *p* = 0.1) and an interaction between muscles and vision (*F*_(1,9)_ = 11.46, *p* < 0.01). The time-constants of TA EO were just smaller than those of Sol EO (*post hoc*, *p* < 0.01), while there was no difference between TA and Sol in the EC condition (*post hoc*, *p* = 0.36). Moreover, there was no difference in the time-constant of Sol between EC and EO (*post hoc*, *p* = 0.1).

At the end of the perturbation cycles (Figure 2D), there was a difference in the mean level of EMG activity between TA and Sol (*F*_(1,9)_ = 11.4, *p* < 0.01). This was due to their different behavior as a function of the visual condition. There was a difference between EC and EO (*F*_(1,9)_ = 16.37, *p* < 0.01) and an interaction between muscles (TA and Sol) and visual condition (*F*_(1,9)_ = 8.9, *p* < 0.05). TA activity at steady state was smaller with EO than EC (*post hoc*, *p* < 0.01), while the level of Sol activity was not affected by vision (*p* = 0.99).

Also in the 10 subjects who performed the EO condition only, TA and Sol reached steady state very quickly (τ =1.50 ± 1.0, TA, and 3.3 ± 6.3, Sol). Overall, there was no difference in the mean time-constants between the two groups (Student’s *t*-test, *p* > 0.3, for TA and Sol muscles, separately analyzed). Therefore, for simplicity and for exploiting repeated-measures tests, we analyzed the findings pertaining to EC and EO behavior only in the group that performed EC before EO.

### Co-Activation Turns to Reciprocal Activation During Adaptation

**Figure 3:**
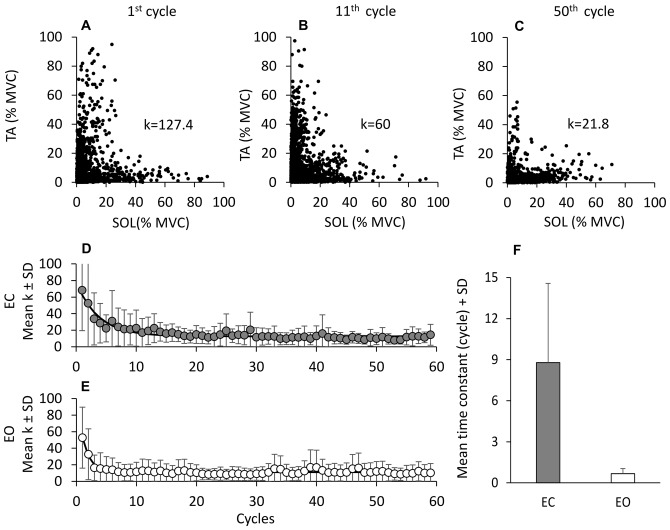
**Reciprocal activation of TA and Sol.** In **(A–C)**, TA activity is plotted against that of Sol for three non-consecutive cycles in one subject to show the progressive decrease in the co-activation pattern. The value of *k* (the TA and Sol co-contraction index) diminishes with cycle repetition. **(D,E)** show the grand means of k, obtained by averaging the *k* of each subject for each subsequent cycle, EC **(D)** and EO **(E)**. The exponential functions fitted to the data points are *y* = 12.4 + 50.4e^−t/4.2^, EC; and *y* = 8.5 + 34.8e^−t/1.2^, EO. **(F)** show the mean time-constants obtained by fitting an exponential function to the data points of each subject and of each condition. The time-constant of the “co-activation” adaptations is much longer EC (gray bar) than EO (white bar).

**Figure 4:**
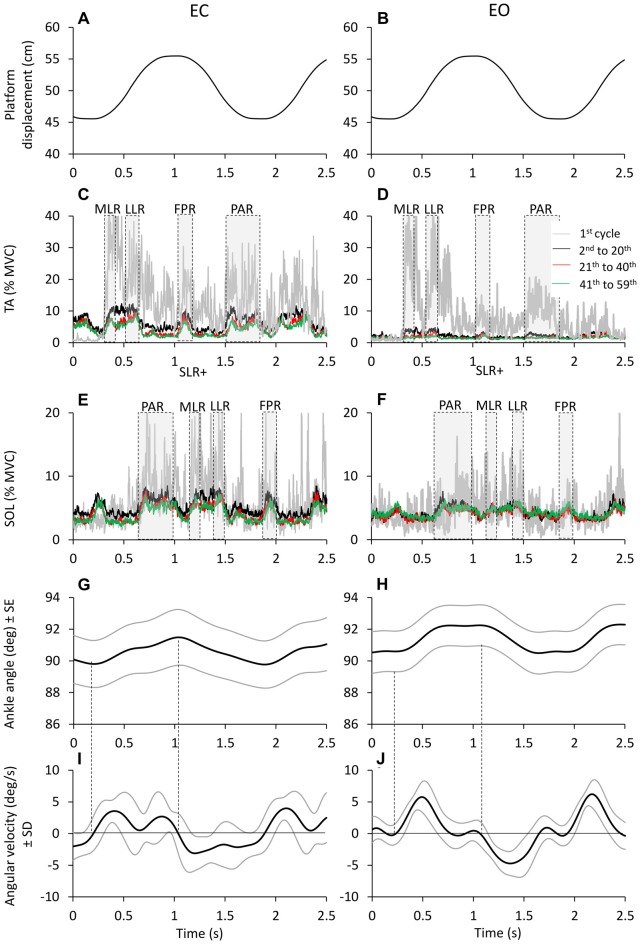
**Time windows of TA and Sol bursts.** Platform displacement **(A,B)**. Mean across subjects of TA **(C,D)** and Sol **(E,F)** EMG of the first cycle (gray traces) 2nd to 20th cycle (black traces), 21th to 40th (red traces) and 41th to 59th (green traces), EC (left panels) and EO (right panels). TA and Sol EMG are almost reciprocal and segmented in different bursts. These bursts rise and decrease sharply as a sign of high synchronization and consistency across subjects. The different time windows of activation of TA and Sol are highlighted by gray dashed boxes. **(G,H)** show the grand averages of the ankle angle changes (subjects and cycles collapsed) and **(I,J)** show the corresponding changes in angular velocity. The black lines refer to the grand mean of ankle angle and angular velocity changes; the gray lines show the standard error for the ankle angular changes and the standard deviation for the angular velocity changes.

Not only did muscle activity decrease over time, but there was also a change in the pattern of recruitment of the antagonist TA and Sol muscles. For all cycles, the TA activity profile was plotted against that of Sol in order to detect any prevalent activation pattern (reciprocal activation or co-activation). Each individual symbol of **(A)** to **(C)** of Figure 3 corresponds to one TA and Sol EMG sample (1000 Hz) recorded during three non-consecutive cycles in a representative subject (the cycles were arbitrarily chosen as examples of the pattern of activity recorded during the first (1st to 20th cycle) and third (41th to 59th cycle) segment of the entire sequence, see Figure 4). The value (*k*) of TA and Sol co-contraction index (see “Materials and Methods” Section) diminished over the cycles. The lower panels (**D**, EC and **E**, EO) show the grand means of *k* of all subjects over cycles, featuring a progressive reduction of the *k* value, therefore a shift from co-activation to reciprocal activation. For each subject, the *k* value was then plotted as a function of the successive cycles and fitted with an exponential function. The average time-constant of the adaptation of *k* (panel **F**) was much larger with EC (8.8 cycles ± 5.8 cycles) than EO (0.6 cycles ± 0.4 cycles; *post hoc*, *p* < 0.001), indicating that the initial co-activation patterns disappeared much more rapidly for EO than EC. Overall, it appears that co-contraction of the antagonist leg muscles promptly subsides with adaptation, more or less rapidly as a function of visual condition, to give way to reciprocal activation.

### Reflex and Anticipatory Responses

TA and Sol bursts showed consistent features across perturbation cycles. The bursts were easily identified in the successive cycles, both EC and EO. This is obvious in Figure 4, showing the grand mean of the rectified EMGs obtained by averaging the traces across subjects and cycles. The gray traces refer to the TA **(C,D)** and Sol burst **(E,F)** recorded during the first cycle. The three superimposed colored traces are the result of averaging the EMG traces of the same muscles recorded during the cycles from 2nd to 20th, 21th to 40th and 41th to 59th (black to green in that order). Activity was much larger in the first cycle (particularly for TA) under both EC and EO condition. The bursts took place within the same time-windows from the beginning to the end of the perturbation cycles. In spite of their diminishing amplitude, TA and Sol profiles were almost reciprocal, underlining the conclusion of the previous paragraph. The four bottom panels of Figure 4 show the grand means of the changes in the ankle angle **(G,H)** and in its angular velocity **(I,J)**. The ankle angle had an average range of about 2°, which would have produced minor, but not negligible changes in length of the muscles around the joint. The angular velocity was overall positive (ankle angle increases and TA lengthens/Sol shortens) during the platform forward translation and negative (ankle angle decreases and TA shortens/Sol lengthens) during the platform backward translation.

**Figure 5:**
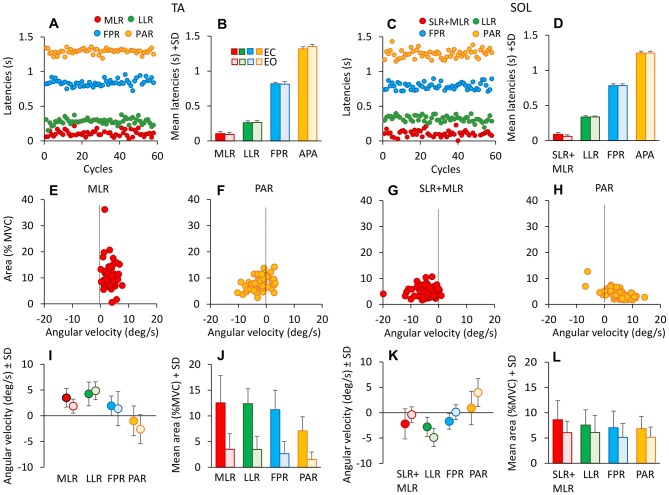
**Latencies of TA and Sol bursts.** These are expressed with respect to the onset of platform forward movement (TA, left panels), and to the onset of backward movement for Sol (right panels). **(A,C)** show the onset latencies for TA and Sol bursts, in one subject cycle by cycle and for all response types (red medium-latency response (MLR) or SLR + MLR, green long-latency response (LLR), blue fall-preventing response (FPR) and yellow proactive response (PAR)). The grand means of the onset latencies of TA **(B)** and Sol **(D)** responses are reported. There are no differences in burst latencies between EC and EO, for both TA and Sol. **(E–H)** show TA (MLR, **E**; PAR, **F**) and Sol (SLR + MLR, **G**; PAR, **H**) burst areas against the corresponding ankle angular velocities for all cycles in one subject EC. TA and Sol reflex responses occur during muscle lengthening (positive ankle velocity for TA, negative for Sol), whereas PAR responses do not, for either TA or Sol. **(I,K)** show the grand means of ankle angular velocities preceding TA **(I)** and Sol **(K)** bursts EC and EO. Velocities are similar between EC and EO, but not across responses. **(J,L)** show the grand means of TA **(J)** and Sol **(L)** response area EC and EO. Bursts are smaller EO than EC for all responses. TA activity diminishes more than Sol activity passing from EC to EO.

Figure 5 shows that these bursts had a remarkably constant latency throughout the successive cycles, in spite of the mentioned changes in size. Latencies were measured from the onset of the forward translation of each cycle for the TA (**A**, one subject) and from the onset of the backward translation for Sol **(C)**. We compared the onset latencies of the four identified TA bursts (EC) across cycles and subjects. ANOVA showed no significant effect of the cycle number on the mean latencies (MLR: *F*_(1,58)_ = 0.75, *p* = 0.9; LLR *F*_(1,58)_ = 1.3, *p* = 0.08; FPR: *F*_(1,58)_ = 1.07, *p* = 0.4; PAR: *F*_(1,58)_ = 1.28, *p* = 0.09). Moreover, the *post hoc* analysis showed no difference in the mean latencies among the first 5 cycles (*p* > 0.05, for all responses). Not only the latencies were consistent within each subject, but they were almost superimposed in all the subjects, as shown by the small standard deviation of the bars reporting the mean latency values for each burst (**B** and **D**). Furthermore, the mean latencies were not different between EC and EO (*F*_(1,9)_ = 0.04, *p* = 0.85).

To account for the variability in the latency of the reflex or PARs, for each subject the standard deviation of the onset latency of the various TA burst (EC) were calculated and compared across responses by the Levene’s test (*F*_(3,36)_ = 8.89, *p* < 0.001). The variability of PAR response proved to be significantly larger than that of the other bursts (*post hoc*, *p* < 0.01 for the four comparisons), while there was no difference across the other responses (*post hoc*, *p* > 0.3).

The mean duration of the bursts across subjects (not shown in the Figure) ranged from about 60 ms (MLR) to about 250 ms (PAR) for TA and from about 65 ms (SLR + MLR) to about 360 ms (PAR) for Sol. For each response, the mean duration of the bursts was not different between EC and EO conditions (*F*_(1,9)_ = 0.075, *p* = 0.79), but was different between muscles (*F*_(1,9)_ = 87.9, *p* < 0.01). There was also an interaction between muscle and vision (*F*_(1,9)_ = 38.3, *p* < 0.01), since durations were generally longer with EO for Sol and with EC for TA, but significantly so only for PAR and FPR response (*post hoc*, *p* < 0.05, for all comparisons).

Figures 5E–H also show the result of the attempt to correlate the size of the bursts to the angular velocity of the ankle angle, in order to identify reflex and non-reflex responses. Some burst proved to be directly connected to stretch of the homonymous muscle, while others were not. The TA bursts at the beginning of each platform forward translation (MLR) were associated with the increase in ankle angle (positive angular velocities in **E**), while other bursts occurred in the absence of consistent positive angular velocity or even with negative velocity (for instance, TA PAR was often present when the ankle angle decreased, corresponding to TA shortening, see **F**). A similar pattern occurred for Sol (**G,H**; note that negative velocities stand here for the reduction in ankle angle, therefore Sol lengthening). The third row (**I** and **K**) summarizes this finding, where the successive points belong to reflex or anticipatory responses within a cycle. Each response occurred at different mean ankle angular velocity, for both TA (*F*_(3,24)_ = 12.88, *p* < 0.001) and Sol (*F*_(3,27)_ = 16.03, *p* < 0.001). In particular, for TA, across subjects and cycles, the mean angular velocities were positive for MLR, LLR and FPR and negative for PAR (there was a difference between the ankle angular velocity for PAR and for the other three responses, *post hoc* *p* < 0.001 for the three comparisons). Figures 5J,L show that burst area was smaller with EO than EC for all TA and Sol responses (*F*_(1,9)_ = 32.35, *p* < 0.001). There was an interaction between muscle and vision (*F*_(1,9)_ = 17.81, *p* < 0.01), since with EO, TA area was much smaller than Sol area, for all responses (*post hoc*, *p* < 0.005). There was a difference between response types (*F*_(3,27)_ = 10.11, *p* < 0.001), and an interaction between the response and vision (*F*_(3,27)_ = 3.17, *p* < 0.05).

**Figure 6:**
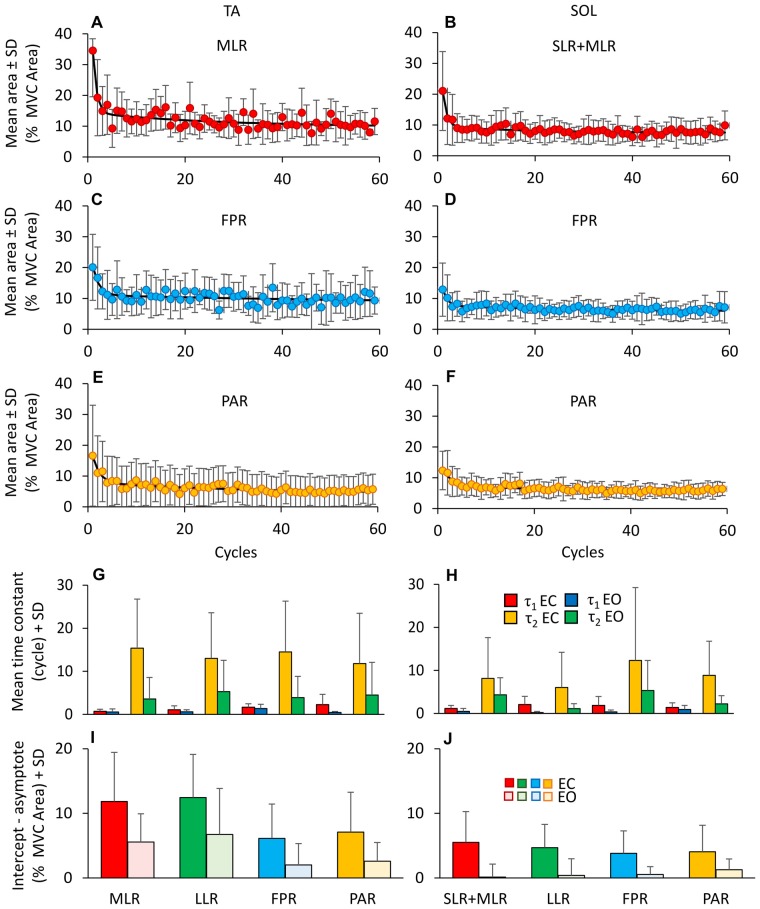
**Adaptation of reflex and anticipatory response. (A–F)** Shows the adaptation of the different TA (MLR, **A**; FPR, **C**; PAR, **E**) and Sol responses (SLR + MLR, **B**; FPR, **D**; PAR, **F**). For TA, the exponential functions fitting the mean burst area are *y* = 9.7 + 20.3e^−t/0.8^ + 4.5e^−t/26.8^ for MLR, *y* = 8.3 + 9.4e^−t/1.3^ + 2.8e^−t/62.6^ for FPR and *y* = 4.3 + 8.5e^−t/1.5^ + 3.6e^−t/32.1^ for PAR. For Sol, the exponential functions are *y* = 7.46 + 11.5e^−t/0.8^ + 2e^−t/17.1^ for SLR + MLR, *y* = 5.7 + 5.4e^−t/1.0^ + 1.9e^−t/29.8^ for FPR and *y* = 5.5 + 5.2e^−t/1.8^ + 2e^−t/22.4^ for PAR. **(G,H)** show the mean time constants obtained by fitting with a double exponential function the TA **(G)** and Sol **(H)** responses EC (*τ*_1_, red bars; *τ*_2_, yellow bars) and EO (*τ*_1_, blue bars; *τ*_2_, green bars). The time constants differ between EC and EO and between TA and Sol. The extent of adaptation in the amplitude of the response bursts **(I,J)** is higher for TA than Sol, higher with EO than EC and different between reflex and anticipatory responses.

Figure 6 shows the change over time in the mean values of the area of the reflex burst (MLR, A and SLR + MLR, B), of the FPR **(C,D)** and of the PAR **(E,F)** for both TA and Sol, EC. For each subject, the decay of the areas of the bursts was described by exponential functions with two time-constants. The mean time-constants for the various responses of all subjects are reported in panels **(G,H)** for TA and SOL, EC and EO. Both TA and Sol showed an initial rapid decrease (*τ*_1_); then, the EMG activity of both muscles continued to decrease more slowly (*τ*_2_). The fitting algorithm did not converge to the exponential model for *n* = 4 bursts of TA PARs (EO condition only) and for *n* = 17 bursts of Sol (comprising both EC and EO proactive and reflex responses), since the amplitude of those bursts proved to be almost constant from the beginning to the end of the perturbation sequence. On averaging the valid time-constant values, *τ*_1_ proved to be shorter than *τ*_2_ (*F*_(1,115)_ = 106.5, *p* < 0.001). All time-constant collapsed, there was a difference between TA and Sol (TA longer than Sol, *F*_(1,115)_ = 6.98, *p* < 0.05) and between visual condition (*τ*_1_ and *τ*_2_ were longer with EC than EO, *F*_(1,115)_ = 59.96, *p* < 0.001). There was an interaction between *τ*_1_ and *τ*_2_, muscles and vision, since vision decreased *τ*_1_ and *τ*_2_ to a larger extent in TA than Sol (*F*_(1,115)_ = 4.89, *p* < 0.05). Vision significantly decreased *τ*_2_ for all TA bursts (*post hoc*, *p* < 0.05 for the four comparisons). All types of response (reflex or proactive) decreased over time with similar time-constants in both muscles (*F*_(3,115)_ = 1.7, *p* = 0.17). This was true both for *τ*_1_ and for *τ*_2_ (interaction between time-constants and type of response, *F*_(3,115)_ = 0.9, *p* = 0.44). With EC, the TA reflex responses exhibited the shortest initial rapid decrease (*τ*_1_, MLR = 0.7 ± 0.5 cycles, TA LLR = 1.1 ± 0.1 cycles) suggesting that the “first trial effect” had vanished already at the 2nd cycle. *τ*_1_ was instead just longer for the proactive than the reflex responses (1.7 ± 0.2 cycles for FPR and 2.3 ± 0.1 cycles for PAR) but not significantly so (*post hoc* *p* > 0.10 for all comparisons).

In order to assess the amount of reduction in the burst area during adaptation, for each burst we calculated the difference between the values of the burst at the second oscillation cycle and the asymptotic value, based on the exponential fit. Figures 6I,J report the amount of reduction calculated for the different responses, for TA **(I)** and Sol **(J)**. A great difference between the value of a response at the second oscillation cycle and the asymptotic value points to a large response adaptation. Conversely, short histogram bars (e.g., **(J)**, EO data) point to minimal amplitude adaptation. Under EO condition, in six subjects, TA responses disappeared in the final perturbation cycles and in two subjects the TA FPR response was never present. The entity of the reduction in amplitude was larger with EC than EO (*F*_(1,9)_ = 8.54, *p* < 0.05) for all the responses and greater for TA than for SOL (*F*_(1,9)_ = 13.88, *p* < 0.01). There was also a difference between type of response (*F*_(3,27)_ = 12.7, *p* < 0.001) and an interaction between type of response and muscles (*F*_(3,27)_ = 13.48, *p* < 0.001). For TA, both under EC and EO condition, the entity of adaptation of the reflex responses (MLR and LLR) was larger than that of the PARs (FPR and PAR; *post hoc*, *p* < 0.05, for all comparisons).

### Head and CoM A-P Behavior During the Entire Perturbation Period

The value of the standard deviation of the entire trace of the head and CoM displacements was calculated (not shown) to get a global index of the segment oscillation amplitude. This mean across-subjects oscillation index was for the CoM: 3.1 ± 0.8 cm (EC), and 2.3 ± 0.8 cm (EO), and for the head: 5.1 ± 2.0 cm (EC), and 2.3 ± 0.7 cm (EO). ANOVA showed an effect of vision on the oscillation indexes (*F*_(1,9)_ = 34.25, *p* < 0.001), a difference between head and CoM index (*F*_(1,9)_ = 7.74, *p* < 0.05), and an interaction with vision (*F*_(1,9)_ = 94.92, *p* < 0.001). On average, the oscillation indexes of head and CoM were not different with EO (*post hoc*, *p* = 0.05), while closing the eyes induced a larger oscillation for head than CoM (*post hoc*, *p* < 0.001).

**Figure 7:**
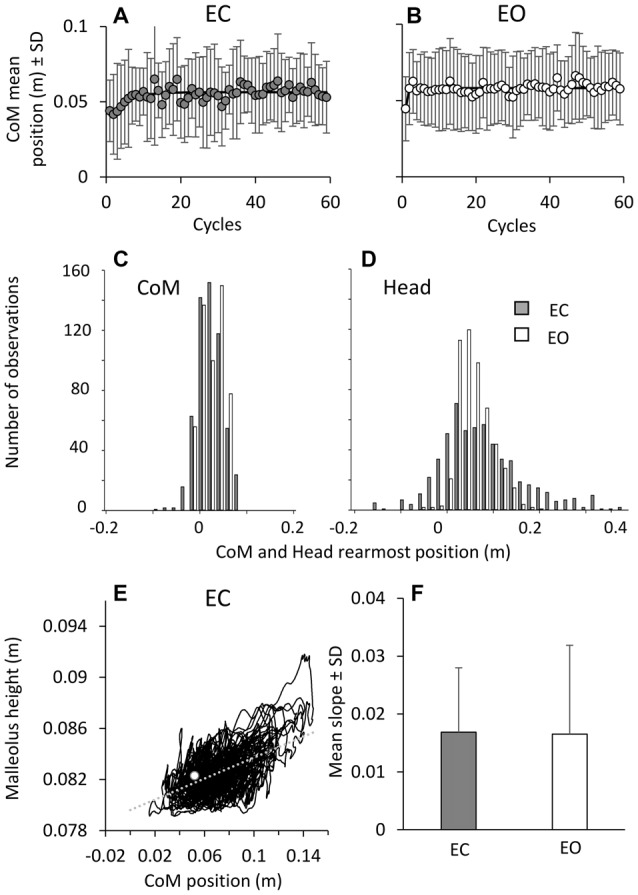
**Changes in CoM positions over time.** During the perturbation cycles, the CoM mean positions with respect to malleolus **(A,B)** exhibited a minor shift forwards, both EC (left) and EO (right). The exponential functions fitted to the mean positions of CoM are *y* = 0.056 − 0.015e^−t/4.4^ in EC and *y* = 0.058 − 0.013e^−t/0.1^ in EO. **(C,D)** show CoM and head backward-most position (all trials and all subjects collapsed). These only infrequently EC (gray bars) and never EO (white bars) bypass the position of the malleolus (backwards negative in the abscissa). **(E)** shows the direct relationship (*y* = 0.04x + 0.08; *r*^2^ = 0.3; *p* < 0.001) between CoM position with respect to malleolus and the height of the marker placed on malleolus in a representative subject under EC condition. The white dot indicates the values of CoM position and malleolus height during quite stance. The mean slope across subjects of the regression line indicating the relationship between CoM position and malleolus height is reported in **(F)** for EC (gray bar) and EO (white bar).

The CoM A-P positions with respect to malleolus are critical to balance, since any translation of the CoM behind the malleolus increases the risk of falling backwards. There was no difference in the CoM position between EC and EO condition (*F*_(1,9)_ = 0.12, *p* = 0.7), both at the beginning (the intercept value of the exponential fit, *post hoc*, *p* = 0.34) and end of the perturbation cycles (the asymptotic value of the fit, *post hoc*, *p* = 0.75; Figures 7A,B). The CoM mean positions **(A,B)** exhibited a moderate shift forwards of about 1 cm with respect to its position in the first cycle, with a time-constant of 3.4 ± 2.9 cycles (EC) and 2.0 ± 1.9 cycles (EO). There was no difference in the time-constant between EC and EO (*t*-test, *p* = 0.2), but a difference in the CoM position between the first cycle and the final cycles (*F*_(1,9)_ = 20.45, *p* < 0.05), both for EC and EO (*post hoc*, *p* < 0.05). After the initial forward shift, the CoM position remained remarkably constant throughout the following cycles with both EC and EO, indicating an overall similar inclination of the body with respect to the ankle joint. The bottom panels show that, on collapsing all trials and subjects, CoM **(C)** and head **(D)** backward-most positions infrequently (EC) and never (EO) bypassed the position of the malleolus. The graphs also show the limited scatter of CoM compared with the larger head data scatter (EC) across subjects and trials. Overall, the control of the CoM position relative to the base of support (critical for preventing backward fall) proved to be effective from the beginning to the end of the perturbation cycles regardless of the major attenuation of the EMG bursts.

The anterior displacement of the CoM was accompanied by an increase in the height of the malleolus marker, as a sign of deformation of the foot arch. The direct relationship between CoM advancement and malleolus height in a representative subjects during EC condition is shown in Figure 7E. Across subjects, the average slope of the line best fitting that relationship **(F)** was 0.019 ± 0.012 (EC) and 0.018 ± 0.016 (EO) (*t*-test, *p* = 0.95), indicating that the malleolus height increased by about 0.2 mm for 1 cm of CoM advancement, both under EC and EO condition.

### Changes in the Temporal Relationship of the CoM Position With Respect to the Moving Base of Support

**Figure 8:**
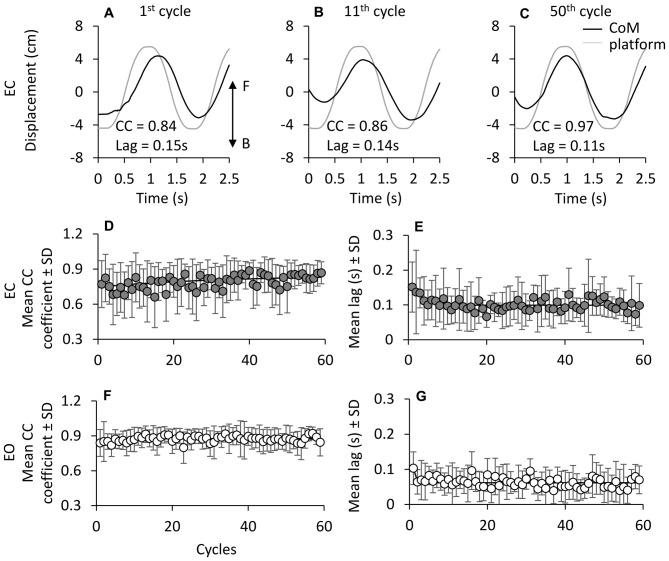
**Cross correlation (CC) between CoM and platform displacement. (A–C)** show CoM (black line) and platform displacement (gray line; both referred to the laboratory space) during three non-consecutive cycles in one subject EC (F, forward; B, backward). **(D,E)** show the grand mean of the CC coefficient at time lag = 0 between CoM and platform displacement traces. CC values slightly increase with cycle repetition under both EC **(D)** and EO **(E)** condition. The exponential functions fitted to the mean CC coefficient data points are *y* = 0.83 − 0.15e^−t/36.3^ in EC and *y* = 0.87 − 0.04e^−t/4.7^ in EO. At the beginning of the oscillation cycles, CoM follows the platform with a lag of about 0.16 s EC, and 0.1 s EO. The time lag between CoM and platform diminishes with cycle **(F,G)** both EC and EO. The exponential function is *y* = 0.10 + 0.05e^−t/2.7^ in EC and *y* = 0.06 + 0.04e^−t/0.5^ in EO.

Figures 8A–C show the progressive time-shift of the CoM with respect to the support base in a representative subject (EC). For all subjects, the CC coefficient between the traces of CoM and platform (Figure 8D) was high from the beginning (*R* = 0.77 ± 0.2, the average of the first cycle across subjects) and tended to increase and level off over time. The mean *R* of the last 5 cycles was 0.84 ± 0.07. The mean time-constant of this change in *R* over time was 11.9 ± 11.5 cycles. With EO (Figure 8E), the CC coefficient was high at the first cycle (*R* = 0.84 ± 0.11) and increased slightly during the successive perturbation cycles (*R* = 0.89 ± 0.06) with a mean time-constant across subjects of 4.8 ± 3.0 cycles. The time-constant of the changes in CC over time was not strongly affected by vision (EC and EO, paired *t*-test, *p* = 0.08).

Figures 8F,G show the average values over time of the CoM-platform time lags, under EC and EO conditions. At the beginning, the displacement of CoM trailed the platform displacement by about 0.16 s with EC and 0.1 s with EO (paired *t*-test, *p* = 0.15). The mean time-lag diminished with cycle repetition to about 0.1 s with EC and 0.07 s with EO (paired *t*-test, *p* = 0.06) with a mean time-constant **(F)** of 7.8 ± 9.7 cycles with EC, and of 4.6 ± 2.9 cycles with EO (paired *t*-test, *p* = 0.33). These lags were small, but significantly different from zero in most perturbation cycles of all subjects (EC, 83%; EO, 80%). Overall, the delay of the CoM with respect to the platform back-and-forth displacement was very limited, as a sign of effective synchrony of body to platform movements, but tended to become in-phase with the platform translation.

**Figure 9:**
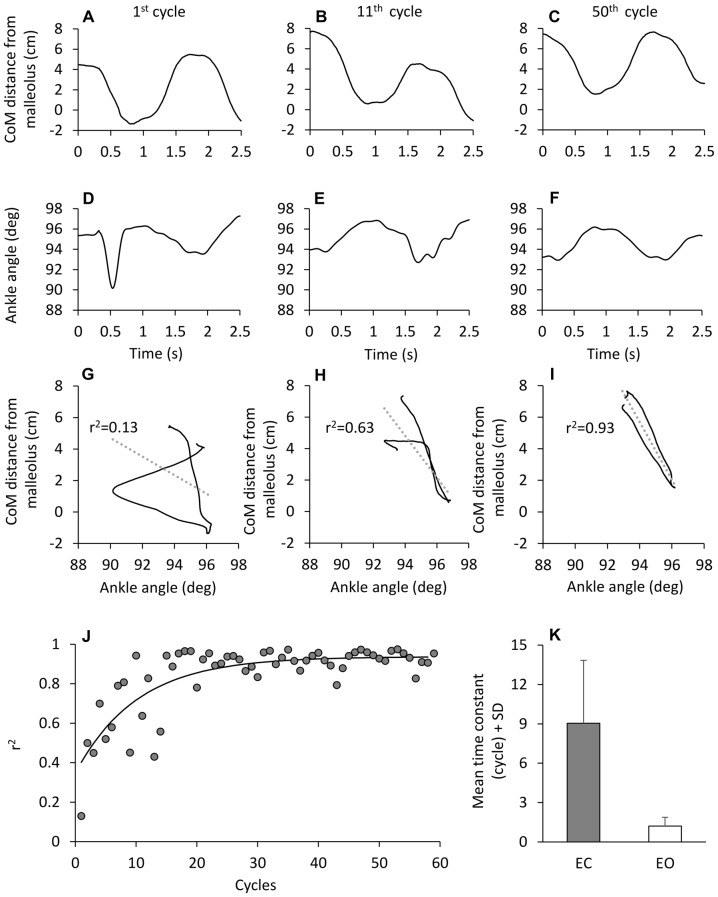
**Time-dependence of the correlation between CoM displacement and ankle angle variation.** **(A–F)** CoM horizontal distance from malleolus **(A–C)** and ankle angle variation **(D–F)** in one subject during three different cycles of perturbation EC. When the CoM distance from malleolus is plotted against ankle angle **(G–I)**, the association between the two traces improves with cycle number. **(J)** show the value of the determination coefficient (*r*^2^) calculated for each cycle EC. The *r*^2^ values increase with cycle repetition with a time-constant of 10.1 cycles (*y* = 0.93 + 0.6e^−t/10.1^). **(K)** show the mean time-constant across subjects, EC (gray bar) and EO (white bar).

The regularity of the CoM cyclic displacement was at the expense of a presumably complex control. When the CoM back-and-forth displacement with respect to malleolus was plotted against ankle angle for each successive cycle (Figure 9), the coefficient of determination of the linear regression (*r*^2^) was initially very low, and improved with time. Then, the “easy” balancing synergy (CoM moving forward when the ankle angle decreased and vice versa), featuring a coefficient of determination approaching unity, was reached with a time-constant of about 10 cycles **(J)**. When the mean time course of *r*^2^ was calculated for all subjects, this finding was replicated. This time-course proved to be much shorter with EO than EC (*post hoc*, *p* < 0.001), as shown in the bottom panel *(K)* of Figure 9, indicating a strong coupling between CoM displacement and ankle angular change with EO from the very beginning.

We note that both the time-constants of the coefficient of determination (*r*^2^) and the time-constant of the co-contraction index **(k)** (Figure 3) were not significantly different (*F*_(1,9)_ = 4.19, *p* = 0.07), suggesting a parallel time-course of both parameters. There was an effect of vision (*F*_(1,9)_ = 28.52, *p* < 0.001), since the time-course was much more rapid for EO than EC. There was no interaction between visual conditions and time-constants (*F*_(1,9)_ = 3.44, *p* = 0.09).

## Discussion

### Progressive Decrease in Leg Muscle EMG Activity

Healthy young subjects underwent repeated and predictable perturbations of stance, featuring continuous A-P translations of the support base calling into action feedback responses and proactive postural adjustments related to the repetitive rhythmic platform displacements (Milner and Franklin, 2005; Franklin et al., 2008). Our aim was to confirm that: (a) leg muscle EMG activity decreases over the perturbation cycles (Schmid et al., 2011) and to assess whether; (b) adaptation is differently modulated for the reflex and anticipatory responses elicited in the leg postural muscles; (c) adaptation implies changes in both amplitude and latency of the response bursts; (d) a relationship exists between EMG response adaptation and critical kinematic variables; in addition, we addressed; and (e) the role of vision on adaptation. We will first discuss the no-vision data (EC), to later consider the effect of vision (EO) on the balancing behavior.

The TA muscle showed in all subjects a progressive decrease in activity, common to both legs (Vieira et al., 2014). The time-constant of this decrease with EC was such that the activity tended to level off to about 30% of its initial value after 15–20 cycles. The time course and the steady-state level of this decrease were not exactly the same in all the subjects, though, as indicated by the variance of the mean data. This could be connected with cycle-to-cycle differences in the balancing behavior, whereby some subjects kept their body straight, trying to counteract the platform displacement, while others allowed a moderate bending of their body by hip and ankle angle changes. In addition, since the stimulation pattern implied a fixed-support strategy and precluded the option of grasping (Maki and McIlroy, 1997), four subjects initially made a short step that was soon corrected. In other subjects, there was a transient phase wherein, after the initial smooth adaptation (Adi-Japha et al., 2008), the balancing performance was possibly disturbed by a gradual forward or backward body lean that required a corrective intervention by modulating the amplitude of the leg muscle bursts.

The Sol muscle also showed a progressive decrease in amplitude, with a time-constant just as like that of the TA, showing a contribution of the Sol to the general process of adaptation. However, contrary to TA, the steady-state EMG level remained elevated in the Sol (about 60% of the initial value). This is likely connected with the need to continuously counteract gravity, since in all subjects and cycles the CoM lay always in front of the malleolus, requiring a continuous plantar-flexor activity. The limited reduction of Sol activity could be also connected to the forward displacement of the CoM (about 1 cm) occurring during the first few initial cycles and persisting until the last cycles (Keshner et al., 1987), which would have increased the gravity torque (Schieppati et al., 1994). In addition, in the course of adaptation, TA activity progressively diminished. Overall, while CoM advancement requires a larger Sol activity to keep the body from falling, the diminishing TA force requires less counter-action by Sol so that, in the end, Sol activity may remain approximately constant.

### The Timing of the Bursts Within Cycles, and the “First-Trial” Effect

Averaging the EMG traces across subjects and perturbation cycles gave a clear-cut profile of the time-varying muscle activity within a cycle. This presented a series of bursts that could be easily encompassed by distinct time-windows. The large TA bursts occurring during the first few cycles had the same latency as the corresponding smaller-amplitude bursts recorded during the following cycles. Hence, the latency of the bursts was independent from the process leading to the attenuation of burst amplitude over time. This was not necessarily predicted, since latency changes *can* occur under a continuous perturbation protocol similar to ours, as after deliberately produced fatigue of TA (Kennedy et al., 2012). One might argue that, in the latter case, the changes in latency take into account the smaller capacity of muscle force production, compelling the neural command to change its timing since changes in force recruitment would be less effective. Without fatigue, the strategy of reducing amplitude but not timing of activation would be adequate.

The latency of the first large TA burst is not different from that of the corresponding bursts of the following cycles. This reduces the probability that the first large burst is a startle reaction (Oude Nijhuis et al., 2010; Campbell et al., 2013), and suggests it to be a true stretch-induced balance-correcting response. McIlroy and Maki (1993) had already emphasized that a response to an unexpected perturbation can contain a compensatory reaction. The fact that subjects wore soundproof earphones also renders unlikely that they reacted with a startle reaction to the noise produced by the platform. The extent to which any “startling” effect of proprioceptive or vestibular origin (Bisdorff et al., 1994; Álvarez-Blanco et al., 2009; Sanders et al., 2015) might have contributed to the large amplitude of the first TA MLR and LLR bursts cannot be settled based on the present data. However, we would note that the body position was closer to the vertical at the time of the first perturbation cycle, as shown by the large ankle angle and the short distance between CoM and malleolus. The greater length of the TA at this time with respect to the subsequent cycles would favor a larger reflex response to the rapid TA stretch induced by the platform forward displacement. As to the bursts following the initial TA reflex responses, namely the so-called FPR and PAR, it seems even less likely that any startle reaction *per se* could have extended its effect to the muscle activity occurring later.

### Adaptation of Reflex and Anticipatory Responses

For almost all cycles of the sequence, the TA burst occurring at the beginning of each platform forward translation had similar latency, in turn similar to the latency of the response to the very first perturbation. This occurred in spite of the burst being triggered by “dynamic” consecutive perturbations, while the first perturbation started from the “neutral” static postural configuration. The amplitude of these TA bursts were always related to the velocity of the increase in ankle angle. These bursts were considered medium- and long-latency stretch reflexes (MLR and LLR), since the angular velocity is a variable appropriate for estimating the velocity of change in muscle length. The latter is the adequate stimulus for triggering the stretch reflex, given the velocity-dependence of the sensitivity of the muscle spindle receptors. The responses occurring at about the anterior platform turn-around points and during the backward translation periods bore instead no relation to the ankle angular velocity. For this reason, these were considered proactive activities and were named FPR and anticipatory PAR, respectively. In this connection, we would point out that continuous sinusoidal rather than single support surface perturbations were employed in this study, and that only leg muscle stretch was considered. Input from other muscles synchronous with some phase of the perturbation displacement might have produced phasic responses in the leg muscles triggered by changes in body segment movements that we have not examined. However, the similarity in the responses’ latencies between EC and EO, two conditions producing different displacements of the body parts, would not readily favor that possibility. Of note, since the ankle angle *decreases* during the period where the PAR occurred (and the TA shortens accordingly), that burst might include the so-called “shortening reaction” (Katz and Rondot, 1978; Bathien et al., 1981; Berardelli and Hallett, 1984), the amplitude of which is known to depend on the central set (Miscio et al., 2001). This would be in keeping with the notion that, during adaptation, PAR can be modulated at cortical level, since the cortex is certainly involved in controlling critical postures (Taube et al., 2006; Maki and McIlroy, 2007; Nardone et al., 2008; Petersen et al., 2009; Tokuno et al., 2009; Bolton et al., 2011; Zwergal et al., 2012; Obata et al., 2014; Fujiwara et al., 2016). It has been recently suggested that the motor cortex confers sophisticated feedback to these responses, thereby potentially participating in their calibration (Pruszynski et al., 2011; Pruszynski and Scott, 2012).

The absolute decrease in amplitude of burst area from the second cycle (in order to exclude the “first-trial effect” from the computation) to steady state was larger for the reflex than for the anticipatory responses. Consequently, the weight of the anticipatory responses *relatively* increased over the series of perturbations. Hence, it can be inferred that adaptation does not prioritize reflex responses, but appropriately weights feedback and feed-forward control. This inference is not in contrast with the conclusion of previous studies (Cenciarini and Peterka, 2006) asserting that, during continuous perturbations, subject’s reliance on information from the stimulus decreases, whereas a sensory reweighting mechanism ensues, tending to favor information that encode body orientation relative to vertical. A difference in the time course of the decrease in burst amplitude between reflex and PARs might potentially reside in muscle mechanical factors (Proske et al., 1993), such as thixotropy. This is dependent on the history of the changes in length and activation of muscle’s extra- and intrafusal fibers. While we would not deny a contribution of these factors to the early adaptation phase, in particular for the ample decrease in amplitude of the reflex response at the 2nd cycle, we would note that the relatively low amplitude and high frequency of the changes in muscle length would not favor a history-dependent change in muscle fiber stiffness in active muscle (Gurfinkel et al., 1997; Altman et al., 2015, in rabbit in Ca^++^-activated single fiber), which might contribute to the overall adaptation process. Further, the latency of the reflex responses did not change over time, whereas thixotropy would be expected to affect latency (Hagbarth et al., 1995). Besides, in our hands, similar changes in leg muscle length occurred during EC and EO perturbations, but the adaptation time course was very different.

### Fast and Slow Adaptation

At first sight, both reflex and anticipatory TA responses decreased over successive cycles almost in parallel, as an indication of progressive general attenuation of the responsiveness of the TA motor pool to all converging excitatory inputs. However, their time-course (both reflex and anticipatory) was fitted by an equation with two exponential terms, in order to comply with the initial rapid decrease (*τ*_1_) followed by a slower decrease (*τ*_2_), and to better address possible differences in adaptation between bursts. The time-constant of the initial rapid decay was the shortest (albeit not significantly so) for the MLR, shaped by the vanishing of the “first-trial effect” (Oude Nijhuis et al., 2010) discussed above. The time-course of the other bursts was also best fitted by two time-constants. A difference was present between *τ*_1_ and *τ*_2_, since all bursts exhibited a rapid decrease during the initial few cycles and all slowly decreased with a similar time-course, until a steady state was reached after 20–30 cycles. We would argue that the first rapid adaptation produces a coarse adjustment of the corrective torques to promptly counteract the risk of falling (Peterka and Loughlin, 2004), while the second slow process would progressively fine tune muscle activity to the new sensory references (Assländer and Peterka, 2014). It is becoming clear that distinct processes can contribute to sensorimotor adaptation (Huberdeau et al., 2015). In a visuomotor adaptation paradigm requiring to overcome perturbation while reaching, one process learns rapidly and depends on an explicit component, the other learns slowly and in an implicit mode (Taylor et al., 2014). It would not be surprising if these processes also applied to balancing, whereby the early rapid and the later slow adaptation would be expression of an explicit and implicit process, respectively.

Parallel analysis of the Sol bursts allowed to identify stretch responses and anticipatory responses as well. As expected, the pattern of Sol EMG activity profile, as obtained by the same averaging procedure as for TA, showed a half-cycle delay with respect to that of TA. This was in keeping with the TA and Sol similar involvement in counteracting oppositely directed platform translations (the Sol reflex burst appeared at the beginning of the backward displacement of the platform, in response to the rapid Sol muscle stretch, and so on for the other response bursts). For Sol, both reflex and anticipatory responses showed a rapid small reduction (*τ*_1_) followed by a further slower reduction (*τ*_2_). The decrease in amplitude from the second cycle to steady state was also similar for the feedback and PARs, but much less pronounced than for TA. It might be suggested that all Sol responses serve the main task of counteracting gravity by being less susceptible to *selective* adaptation. Also in a different context, task- and context-related changes were more clearly present in the TA than in the Sol muscle (Schieppati and Nardone, 1995).

### Relationship Between Muscle Activity and Kinematics During Adaptation

The constant latencies of the response bursts matched the substantial invariance of the CoM, which is kept within safe margins from the beginning. Possibly, this consistency would be guided by the need to keep stable the CoM that is the important variable for balance control (Safavynia and Ting, 2013; Welch and Ting, 2014). The progressive decrease in muscle activity (particularly so in the case of TA) would not alter the critical time-relationships between biomechanical body parameters and instantaneous position of the continuously translating support base.

At the beginning of the perturbation cycles, the coordination between CoM and ankle joint angle was very poor, and gradually achieved larger determination coefficients. Other body muscles (not recorded here) must therefore be recruited to help keep CoM stable at the earliest cycles, compensating for the poorly controlled ankle angle. Cappa et al. (2008) attributed to the upper limbs (free to move) an anticipatory role in counteracting perturbations similar to ours. In our case, subjects’ arms were deliberately kept crossed so that only small not-monitored motions would have minimally affected the CoM displacement (Assländer and Peterka, 2014). Maki and McIlroy (1997) have reviewed the role of limb movements in maintaining upright stance, and suggested that they are substantially finalized in spite of their apparently chaotic production (Corbeil et al., 2013). In a sense, our protocol “forced” the leg muscles to compensate for absence of assistance of the upper limb motion.

Co-contraction around the ankle joint occurs during tasks such as standing on one leg, balancing on an unstable platform or reacting to rotation of a support surface, or walking on a narrow beam (Diener et al., 1983; Keshner et al., 1987; Nielsen and Kagamihara, 1992; Geertsen et al., 2013) or after fatigue (Kennedy et al., 2012). However, co-contraction is not a rule under different critical postures (Sozzi et al., 2013). In our protocol, TA-Sol co-contraction was present during the initial few cycles, and then gradually vanished. Interestingly, the changes over time of the coefficient of determination of CoM displacement vs. ankle angle increased exponentially toward *r*^2^ ≅ 1 with a time-constant almost equal to that of the TA-Sol co-contraction index, suggesting that co-activation can be a major disturbing factor. This feature, whereby higher co-contraction is associated with poor performances, and lower co-contraction with good performances, seems to be a general phenomenon in motor adaptation (Thoroughman and Shadmehr, 1999; Osu et al., 2002; Cordo and Gurfinkel, 2004). Following Osu et al. (2002), we would suggest that increased stiffness is a sign of inaccurate knowledge of the task, and that this subsides as learning builds up. At steady state, co-contraction is almost absent, and riding the platform becomes smoother and more coordinated while stability of the CoM is maintained.

The slight surge in height of the malleolus (about 0.2 mm per cm of CoM advancement) gives evidence of a significant deformation of the foot arch as the CoM of the body moves forward. As shown by Wright et al. (2012), such deformation would contribute to a change in ankle joint angle (<1°) that accompanies CoM advancement. Regrettably, we are not in the position of dissecting out the role in this event of the change in torque over the ankle joint or of any potential activation of plantar foot muscles or toe-extensor muscle (Schieppati et al., 1994), since no electrodes were fixed to the foot plantar surface or dorsum as in Schieppati et al. (1995). On the one hand, we can only argue that plantar foot muscle activity likely occurred when the CoM moved ahead of the malleolus, and that its activity would parallel that of Sol given that these two muscles are agonist and co-active during backward platform translations (Schieppati et al., 1995). On the other hand, the strong similarity of the slopes of the lines best fitting malleolus height and CoM position with EC and EO (two conditions exhibiting large difference in TA activity) would point to a role for the mechanical constraint rather than for the TA EMG adaptation.

### The Effect of Vision on the Adaptation of the Balancing Behavior

When the same perturbation protocol was administered with EO, clear-cut differences and invariances were observed with respect to EC. As a preliminary note, we would remind here that the EC and EO tests were not performed in a randomized order. The fact that subjects under EO condition were familiar with the protocol, having previously performed the same protocol with EC, might have influenced the adaptation pattern (Pai and Bhatt, 2007; Patel and Bhatt, 2015). However, in *ad hoc* experiments performed in a different group of matched subjects, EO administered first produced a very rapid adaptation, equivalent to that observed in the main subject group. For this reason, we are confident that the very rapid adaptation with EO did not depend critically on the earlier EC trial.

The time-constant *τ*_1_ of the profile of the TA bursts with EO was very short and not significantly different from EC. This suggests that the proprioceptive control predominates for the responses to the first perturbation cycle also when vision is available. The time-constant *τ*_2_ was also short with EO (significantly shorter than EC). In some subjects and bursts (especially in the case of the FPR response), the fitting algorithm did not even converge to an exponential curve, since the EMG activity was low from the very beginning. Therefore, we would put forward the notion that the effect of vision does not only consist in speeding up the adaptation process. It would also consist in a generalized down-modulation of the excitability of the motor neurons of the postural muscles, which are the final common pathway for both reflex and proactive activation (Schieppati et al., 2002; De Nunzio et al., 2005; Schmid et al., 2011).

The time-constant *τ*_1_ (EO) was about one cycle. Hence, with vision, experience of one single cycle is enough to reprogram the balancing behavior in the successive cycles. With EO, the adaptation rate may almost be the sole consequence of the vanishing of the “first-trial effect” (Allum et al., 2011). In a different study from this laboratory, an equally similar rapid adaptation in the balancing strategy and EMG activity was observed when passing from EC to EO condition *during* a series of perturbations similar to that used here (De Nunzio and Schieppati, 2007), generalizing the effect of vision beyond the first-trial effect. Also the stabilization of a standing posture (as measured by the reduction of body sway) on adding vision or haptic information is fast (Sozzi et al., 2011; Blouin et al., 2014). This suggests that addition of vision can very rapidly entrain a low-motion, low-activity balancing pattern.

The prediction that, with adaptation, the final level of TA activity with EC at steady state could have reached the level of activity observed with EO, signifying the attainment of a more energy-efficient mode of balancing, was not verified. The absolute level of TA activity at steady state was in fact substantially larger EC than EO. Therefore, even if TA activity *can* decrease to very low levels, absence of vision precludes what would seem an easily attainable task. On the contrary, Sol activity at steady state with EO remained almost similar to that recorded for EC, indicating major differences in the functional outcomes of adaptation according to muscles having different purposes, as mentioned above. With EO, the TA-Sol co-activation index was also low from the beginning and rapidly diminished further in the following cycles. Also, the coordination between CoM and ankle angle under EO promptly reached a high value. Moreover, the time lag between CoM and back-and-forth movement of the platform, which was just smaller for EO than EC, reached steady state very soon. Conversely, the mean position and the extent of back-and-forth displacement of the CoM were similar across cycles between EC and EO, both in amplitude and rapidity to attain a steady state. Therefore, vision does not confer any particular stability to the body’ CoM. In contrast, the back-and-forth displacement of the head was remarkably reduced by vision, in keeping with previous findings (Buchanan and Horak, 1999; Corna et al., 1999; Schieppati et al., 2002; De Nunzio and Schieppati, 2007; Schmid et al., 2007). Therefore, we would interpret the head stabilization in space with EO as the direct consequence of vision, an input that critically helps defining the reference frame for balance control by conferring the nervous system information about the environment (Dokka et al., 2009; Isableu et al., 2010; Joseph Jilk et al., 2014). Since it is the head that bears the eyes, the rapid head stabilization in space with EO and minimization of its peak-to-peak displacement would mainly contribute to minimizing the changes in visual flow connected with the platform displacement (Schmid et al., 2008; Dokka et al., 2009; Kiemel et al., 2011). In the absence of the possibility of exploiting vision, our brain accepts some extra activity in the postural muscles.

Perhaps, minimization in the metabolic cost has a neural *computational* cost. While the process of adaptation put in action under EC seems to be insufficient for completing this minimization, the neural computation would be easily afforded with vision. Moreover, with clear perception of environment and body movement in space (Gresty and Bronstein, 1992), the brain need not previously experience repeated cyclic events, but uses sort of a “default” strategy as a substitute of a costly adaptation process. It is tempting to assume that the brain without vision undergoes a complex trial-and-error course, whereby balancing improvements are gained from repeated practice by processing down-up information, whereas vision allows an almost immediate shift from a down-up to a top-down control strategy. Admittedly, the possibility of generalizing these inferences is limited by the absence of concurrent recording of the EMG activity of other muscles, potentially relevant in the process of the bodily adaptation to the continuous perturbations. Further studies are needed for detailing the contribution of (rapid or slow) adaptation of other muscle groups in this task.

## Concluding Remarks

Taken together, the findings show that adaptation occurs when a subject’s balance is challenged by repeated perturbations. Under these circumstances, the underlying sensorimotor adaptive processes would include the initial collection of information about the task to handle (among others, from the moving support surface and the position of the CoM), determine how to improve the performance (e.g., reducing redundant muscle activity), to then implement these changes as enhanced motor control performance by repeated trial and error procedure. Contrary to what happens with a self-paced movement, under this balance task condition, the initiator of the process is the initial exposure to an external stimulus (the onset of the series of platform oscillation cycles). This implies reflexive stability-enhancing countermeasures at the same time as it produces an important volley of afferent information about the bodily events. Progressive improvements in balancing behavior, underscored by reduced postural muscle activity and better intersegmental coordination, supervenes from repeated practice.

The ensuing adaptation process selectively affects different neural circuits, since TA activity shows a rapid followed by a slow reduction, while Sol shows a rapid minor adaptation followed by no-reduction in activity (prioritizing antigravity action). Both feedback and feed-forward controls are gradually tuned: yet, the smallest reduction is observed for anticipatory responses (featuring a partial shift from feedback to feed-forward control). Further, any initial TA-Sol co-contraction subsides with the repetition of successive cycles passing from a stiffening strategy to antagonist muscles reciprocal activation. With vision, almost no adaptation is observed after the first few cycles, and the head reduces its motion already at the 2nd perturbation cycle regardless of the persistent CoM back-and-forth displacement.

We therefore suggest that adaptation serves the purpose of: (a) reducing muscle activity and co-contraction while allowing continuous CoM back-and-forth displacement in compliance with the platform translations (*“effort-reducing” adaptation*); (b) improving body segment coordination, reducing over time the number of unnecessary movements (*“entropy-reducing” adaptation*); (c) diminishing head back-and-forth displacement when eyes are open, allowing stable vision (*“visual-field stabilizing” adaptation*). We would also note that standing and balancing on the continuously translating platform shares several features of voluntary control: subjects are aware of the critical stance condition particularly in the absence of visual input, clearly perceive the body displacements accompanying platform displacements, and produce anticipatory adjustments or react to it with correcting counter-movements (Ouchi et al., 1999). Hence, the role of the sensory input in maintaining equilibrium may not be different from the role sensory input plays in directing and correcting voluntary movements of the upper limb (Patla et al., 2002; Soto et al., 2006; Crevecoeur et al., 2012; Hemami and Moussavi, 2014). This ultimately suggests that the cerebral cortex may be the site of the sensorimotor integration and reweighting processes underlying balance control (Taube et al., 2007; Sumner and Husain, 2008; Honeine et al., 2015) and suggests that a similar repertoire of basic mechanisms lies behind skill acquisition in voluntary and postural tasks (Elion et al., 2015).

Postural adaptations occurring from day to day have been reported (Tjernström et al., 2010b; Nardone et al., 2015) and suggested to underpin the rehabilitation effects of repeated perturbations. Predictable balance perturbations by sinusoidal translations have been successfully employed for rehabilitation of balance in selected patient groups (Nardone et al., 2010, 2014) including vestibular patients (Corna et al., 2003), who may badly behave on the platform when not compensated (Buchanan and Horak, 2001–2002). Interestingly, the complex processes of adaptive behavior in a balancing task are able to produce true learning, the uniqueness of which would be to prepare the body to counteract more general features of platform motion (Van Ooteghem et al., 2008; Kanekar and Aruin, 2015). The analytical approach described here could be easily applied to patients with balance problems of different nature, as for instance patients with Parkinson’s disease, who may well show adaptation problems contributing to their balance dysfunction (Schieppati and Nardone, 1991; De Nunzio and Schieppati, 2007; Weissblueth et al., 2008; Nanhoe-Mahabier et al., 2012; Paul et al., 2013; Schoneburg et al., 2013), and patients with stroke (Kitago and Krakauer, 2013) or spinal cord injury, in which co-activation of TA and Sol contribute to impaired balance and walking ability (Beauparlant et al., 2013; Manella et al., 2013).

## Author Contributions

MS and AN conceptualized the study and designed the experiments. SS performed the experiments. SS and MS performed the data analysis. All the authors made contributions in drafting the manuscript and have approved the final version.

## Funding

Supported in part by “Ricerca Finalizzata” grants (RF-2010-2312497, RF-2011-02352379) from the Italian Ministry of Health to AN and MS, and by “PRIN” grants (2009JMMYFZ and 2010MEFNF7) from the Italian Ministry of University to MS.

## Conflict of Interest Statement

The authors declare that the research was conducted in the absence of any commercial or financial relationships that could be construed as a potential conflict of interest.

## Abbreviations

A-P: antero-posterior
CC: cross-correlation
CoM: center of mass
EC: eyes closed
EO: eyes open
FPR: fall preventing response
LLR: long latency response
MLR: medium latency response
PAR: proactive response
SLR: short latency response
Sol: Soleus
TA: Tibialis Anterior.
